# The effect of self-efficacy and self-set grade goals on academic outcomes

**DOI:** 10.3389/fpsyg.2024.1324007

**Published:** 2024-03-28

**Authors:** Katrin Saks

**Affiliations:** Institute of Education, University of Tartu, Tartu, Estonia

**Keywords:** goal setting, self-efficacy, grade goals, learning outcomes, structural equation modeling

## Abstract

**Introduction:**

Numerous motivational research have clearly demonstrated the critical role of self-processes in goal setting, self-regulated learning, and learning outcomes. However, studies have emerged that present conflicting findings regarding the relationship between goal setting and self-efficacy and how it affects academic performance. Based on the theories of goal setting and self-efficacy, the purpose of this paper is to assess the predictive power of self-efficacy and the mediating effect of self-set grade goals on learning outcomes.

**Methods:**

As part of the online course, an exploratory study was conducted with a sample of 160 university teacher training students. Data on self-efficacy were collected with the MSLQ and analyzed with confirmatory factor analysis. Correlation analysis explored the relationships between motivational factors, grade goals, and learning outcomes. To test the models, structural equation analysis was conducted to predict the effect of self-efficacy on self-set grade goals and learning outcomes.

**Results:**

The results showed the predictive effect of self-efficacy on expected and minimum grade goals and the mediating effect of expected grade goal on learning outcomes.

**Discussion:**

The study highlights the influence of motivational factors on goal setting in the context of online learning and provides insight into the predictive effect of self-efficacy on learning outcomes.

## 1 Introduction

Despite the many advantages of asynchronous e-learning, such as learning regardless of time and place, learning at your own pace, personal approach, and considering the individual needs of the learner, e-learning also entails risks that can cause interruptions in studies. Some of the most important of these are ensuring the continuity of learning, developing an independent, responsible attitude, and taking responsibility for one’s learning process (learner autonomy and agency). Low learning motivation and lack of learning skills, including self-regulation and goal setting, often lead to course interruption and dropout (Figueroa-Cañas and Sancho-Vinuesa, 2019). COVID-19 was accompanied by large-scale digitalization of the learning process, which put both learners and teachers to the test. Although increased e-learning opportunities are recognized as a way out and an opportunity to continue studies when contact learning is no longer possible, it may increase interruptions and dropouts (Naylor and Nyanjom, 2020) if the necessary learning skills and prerequisites are unavailable.

Several studies have indicated the students’ perceptions of the extrinsic reasons for dropout like the unsatisfactory online course design and quality (Yang et al., 2017; Joo et al., 2018), teachers’ insufficient digital teaching skills (Cabero-Almenara et al., 2020), or their inability to engage learners and teach successfully in a digital learning environment (Xavier and Meneses, 2021). Many studies (e.g., El Said, 2017; Tsai et al., 2018), however, show the students’ poor performance proficiency, inability to organize and regulate their learning process, and take responsibility for their learning outcomes. The latter is the key to the development of learner autonomy and agency, which, in line with basic self-regulated learning skills, pave the way to the development of lifelong learners (Saks, 2016).

It is now well established in a variety of motivational studies that self-processes play a crucial role in self-regulated learning, goal setting and learning outcomes. However, literature has emerged that offers conflicting findings regarding the relationship between self-efficacy and goal setting and their impact on academic achievement, a gap that this study aims to fill.

## 2 Theoretical framework

The concept of self-regulated learning (SRL), initially originating from the socio-cognitive perspective (Pintrich, 2000; Zimmerman, 2000), includes cognitive, metacognitive, motivational, affective and contextual factors. The current research draws on the general cognitive view of and the definition of self-regulated learning by Pintrich (2000) according to which self-regulated learning is an active, constructive process where learners set goals for their learning, monitor, regulate and control their cognition, motivation, and behavior, guided and constrained by their goals and contextual features on the environment. Research has shown that learners with better self-regulation skills achieve better results in the learning process (Saks and Leijen, 2018) and are more aware of and committed to their learning goals and how to move toward them. They are able to choose and implement the most effective learning strategies, and are able to find and use help in case of problems or obstacles. Describing the SRL perspective, Pintrich (2004) extracted four general assumptions that most models share – (1) active, constructive assumption, (2) potential for control assumption, (3) mediators between personal and contextual characteristics and actual achievement or performance assumption, and (4) goal, standard or criterion assumption. SRL models assume the presence of goal or criterion against which the learning process is assessed. In the regular process of SRL, the individual sets goals, monitors his progress toward these goals, and adapts and regulates his cognition, motivation, and behavior to reach these goals (Pintrich, 2004). In the student approaches to learning (SAL) models (e.g., Biggs, 1993), the goals are distinguished into extrinsic (linked to the surface learning approach) and intrinsic goals (linked to the deep learning approach) based on motivation and strategies for learning. In regular learning situations, however, the learner flexibly combines and adapts the goals and strategies according to the situational and contextual changes, before starting with the task as well as during the process (Saks and Leijen, 2019).

The goals the learner sets for his learning are related to their expectancies for success, their subjective task values, and cost translated to time, effort, and loss of valued alternatives (Eccles and Wigfield, 2002, 2020). Expectancies for success, or personal efficacy, as Bandura (1977) called it, is an individual’s beliefs about how well they will do on an impending task (Eccles and Wigfield, 2020). According to Zimmerman and Bandura (1994), moving toward goals takes place mainly through reflexive processes (e.g., perceived self-efficacy, and effort). The higher the learner’s self-efficacy, the higher the goals and the stronger the commitment to the goals (Locke and Latham, 2002; Morisano, 2013). Self-efficacy affects the level of goals set by the learner, as well as the effectiveness of strategies and responses to failures (Erez and Judge, 2001). Failure can reduce self-efficacy and lead to abandonment or setting lower goals, but those with high self-efficacy respond to setbacks with greater effort and commitment. Studies have shown that self-efficacy affects both effort and persistence, mediators of performance goals (e.g., focus, choice of appropriate learning strategies), and performance in learning tasks (Morisano, 2013). Self-efficacy holds two kinds of expectancy beliefs – outcome expectations (beliefs that certain behaviors lead to desired outcomes) and efficacy expectations (beliefs about effective performance to produce the outcome) (Wigfield and Eccles, 2000). According to Bandura (1997), individuals’ efficacy expectations determine goal setting, activity choice, willingness to expend effort, and persistence.

### 2.1 Goal setting

Goal-setting theory is a theory of motivation that explains what causes some people to perform better on learning or work-related tasks than others. The term goal is defined in goal-setting theory as the object or aim of an action (Locke and Latham, 1990). Goal setting is operationalized as a state that involves identifying the desired outcomes and developing a plan to achieve them.

Goal setting has a prominent role in social-cognitive learning models. It is one of the prerequisites for successful self-regulated learning. Encouraging learners to set goals is used widely to promote behavior change (Epton et al., 2017). When the goals are specific (Locke and Latham, 2002), attainable (Brunstein, 1993), optimally challenging, and relatively close at hand (Koestner et al., 2002), they have a strong impact on both learning behavior and performance. Goals with specific performance standards activate self-evaluations of progress and enhance motivation more than general goals. Short-term goals enhance motivation better than long-term goals, also the goals learners perceive as difficult but attainable than goals that are very easy or overly difficult (Schunk and DiBenedetto, 2021). In their systematic literature review, Locke et al. (1981) found that according to goal theory, difficult objectives lead to better performance than easy goals, notwithstanding their lower possibility of being fully reached. On the other hand, expectancy theory predicts the reverse – a positive relationship between expectancy and performance, provided all other factors remain equal. Therefore, low anticipation ratings may indicate that a participant was not intending to give it their all, while high ratings would indicate the reverse. A fictitious positive correlation between expectancy and performance would result from this.

The goal-setting theory (Locke and Latham, 2002) emphasizes the role of difficulty and specificity of performance goals, learner persistence, and commitment, plus praise and feedback as supporting mediators in accomplishing the set goals. Based on the earlier studies by Morisano et al. (2010) formulated the four mechanisms through which goals affect performance. First, goals direct cognitive and behaviorally attention and effort toward goal-relevant activities; second, high goals energize and lead to greater effort than low goals; third, demanding goals prolong effort and increase persistence; and fourth, goals have an indirect effect on learner action by leading to the discovery, and utilizing task-relevant knowledge and strategies.

Successful goal setting and moving toward goals may be affected by several conditions. Besides the specific, attainable, and challenging nature of the goals, other factors that play an important role in ensuring the progress toward the goal achievement include learner motivation, positive expectations, and realism (Perrone et al., 2004). Koestner et al. (2002) add the importance of a detailed implementation plan (time schedule and backup plan included), and Karakowsky and Mann (2008) consistency. If setting goals and successfully moving toward them supports self-efficacy, a person begins to set higher goals but also creates higher expectations for success (goals beget goals). Although goals do not affect motivation directly, they are considered to be strong motivators. Goals support focus and learner’s efforts toward task success. A discrepancy between the goal and perceived progress can motivate learners to increase the necessary effort and persistence (Schunk and DiBenedetto, 2021).

Goals have been addressed and classified from different perspectives. The intrinsic and extrinsic goals based on the SAL model (Pintrich, 2004) were mentioned above. Goals may also be classified according to the temporal perspective – long-term goals and short-term goals in the context of agency (Biesta et al., 2015; Leijen et al., 2020), proximal and distal goals in the context of self-efficacy theory (Bandura and Schunk, 1981), and outcome/performance goals and mastery/behavioral/learning goals in the self-theory (Dweck, 2000) and goal-setting theory (Locke and Latham, 2006). Eccles and Wigfield (2002) mapped the areas where goal orientation, operationalized as a trait, has been widely explored in traditional learning environments.

Grade goal (or self-set grade goal) is a type of goal that an individual defines as a desired standard for grade outcome. Grade goals are considered to be linked to academic performance (Talsma et al., 2023); however, their effect has been reported to be rather low (Dekker et al., 2021). The relationship between grade goals and academic performance depends on students’ personality traits, for example, core self-evaluations (Bipp et al., 2015) that integrate traits such as self-esteem, generalized self-efficacy, locus of control, and emotional stability. Previous studies have listed numerous variables that mediate self-set goals and academic outcomes to a greater or lesser extent, e.g., cognitive ability, learning skills, self-efficacy, emotions, etc. Academic outcomes have been found to be negatively related to making goal choices based on parental expectations and coping with failure, but positively related to learner self-concept. In addition, students’ goal choices mediate the effect of their grade goals on exam performance (Morisano, 2013).

The conditions of e-learning add special features that the traditional learning context inevitably ignores. Therefore, given the current global health, economic, and security situation, where e-learning is often the only way to pursue education, it is crucial to understand the impact of goal setting on learning behaviors in the e-learning context.

### 2.2 Self-efficacy

In 1977, a Canadian-American psychologist, Albert Bandura defined self-efficacy as an individual’s belief in their capacity to act in the ways which are necessary to reach their goals. According to his social-cognitive theory, human actions are influenced by the interplay of personal, environmental, and behavioral factors. The learner who feels competent in the learning process can make better use of the opportunities offered by the environment (e.g., classroom) and modify their behavior accordingly. This is driven by the desire for the sense of agency realized by setting goals and implementing strategies to attain them. This agentic perspective is affected by the sense of self-efficacy resulting from evaluative and goal-oriented self-reflection (Schunk and DiBenedetto, 2021).

Self-efficacy can be divided into two categories: general and task-specific self-efficacy. An individual’s assessment of their capacity to function in a range of diverse circumstances is known as general self-efficacy. It evaluates a broad and steady sense of personal competence to deal with a variety of tense situations (Scherbaum et al., 2006). According to Eden (1988), general self-efficacy is a stable trait-like attribute, while specialized self-efficacy is a relatively flexible independent variable. Task-specific self-efficacy, as assessed in a particular domain, looks at how someone feels about their capacity to carry out situation-specific actions. Perceived task-specific self-efficacy, which is contingent upon context and situational demands, is measured to obtain a relevant picture of an individual’s confidence in their capacity to perform a certain task or skill (Bandura, 2006). In a range of tasks and contexts, general self-efficacy has a positive impact on task-specific self-efficacy (Sherer and Maddux, 1982) – when an individual has high self-efficacy across several settings and activities, it typically translates to circumstances that are exclusive to that particular activity (Chen et al., 2001).

Pintrich (1991) do not view self-efficacy as a static trait but as varying across different performance domains. Pintrich (1991) distinguished two aspects of expectancy in the scale of self-efficacy for learning and performance. First, expectancy for success [or personal efficacy (Bandura and Watts, 1996) or outcome expectations (Bandura, 1997)] is related to task performance and refers to performance expectations or individuals’ beliefs about how well they will do on an upcoming task (Eccles and Wigfield, 2020). Second, self-efficacy [or efficacy expectations (Bandura, 1997)], which includes an individual’s judgments about their ability to accomplish a task and their confidence in their skills to perform the task, is a self-appraisal of their ability to master a task. However, validity studies have rarely shown these two aspects to constitute distinct factors (Lee et al., 2020). In the current study, we are focusing on the learners’ situation-specific self-efficacy while they self-report their self-efficacy and expectancies for success in the context of a certain online course.

As a motivational construct, self-efficacy is key to promoting student engagement and learning (Linnenbrink and Pintrich, 2003). However, it is not always easy to understand its role in the learning process. According to Bandura (1977), self-efficacy expectations rely on four sources of information. Performance accomplishments (or inactive mastery experience) are based on the learner’s previous successful experience (Honicke et al., 2020). Repeated successes create an expectation of efficacy, which reduces the negative impact of failure (Alqurashi, 2016). Vicarious experience, where a learner observes others performing an activity successfully, is based on social comparison. The vicarious experience becomes critical in the context of e-learning, where learners tend to remain isolated and have to deal with their tasks alone. To avoid this, learning tasks and activities should be created that require cooperation, sharing, peer assessment, and mutual feedback (Korucu-Kış, 2021). The third source of information, verbal persuasion (or social persuasion) is mostly used in the form of feedback (Lam and Chan, 2017). When encouraging learners to measure their success in terms of self-improvement rather than triumph over others (Bandura, 1997) may lead to higher self-efficacy. Unrealistic feedback, however, may lower learner self-efficacy (Alqurashi, 2016). The fourth source of information, physiological states, refers to negative arousals like stress and anxiety, which have a direct negative effect on learner self-efficacy. While the previously mentioned sources of information have impact on learner self-efficacy, then self-efficacy leads to goal choices (Vincent et al., 2021), effort and persistence (Thompson et al., 2022), behavioral, cognitive and motivational engagement (Linnenbrink and Pintrich, 2003), and these in turn result in academic achievement and self-regulation (Andres, 2020; Hayat et al., 2020). Even though most of the studies listed above were conducted in the context of traditional classrooms, similar sources have been identified in online learning environments (Lin et al., 2013).

The interactions between self-efficacy and goals are not always clear. Morisano (2013) listed the mediators – attention, effort, persistence, and task strategies; and moderators – commitment, feedback, and knowledge and skills, which all may influence an individual when moving toward their goals. The higher the learner’s self-efficacy, the higher the goals they set and the greater their commitment to their fulfillment. This, in turn, supports the growth of self-efficacy.

The purpose of this work is to evaluate the predictive power of self-efficacy and the mediating effect of self-set grade goals on learning outcomes. The following guiding research questions were formulated for this work:

(1) What are the psychometric properties and indicators of the factor of self-efficacy?

(2) What are the indicators of the learners’ self-set grade goals?

(3) What is the effect of self-efficacy on self-set grade goals – ideal, expected, and minimum acceptable?

(4) To what extent do self-set grade goals – ideal, expected, and minimum acceptable – mediate the effect of self-efficacy on actual learning outcomes?

Proceeding from the theory the following hypotheses were addressed:

*H*1: Self-efficacy has a positive effect on learners’ self-set grade goals – ideal, expected, and minimum acceptable.

*H*2: Minimum acceptable is the self-set grade goal which most precisely predicts the actual learning outcomes (summative course grade).

*H*3: Self-efficacy has an indirect effect on learning outcomes.

In order to answer the research questions, an exploratory study was conducted with teacher training students of a higher education institution within the framework of an online course.

### 2.3 Hypothesized models and their theoretical justification

**Figure 1:**
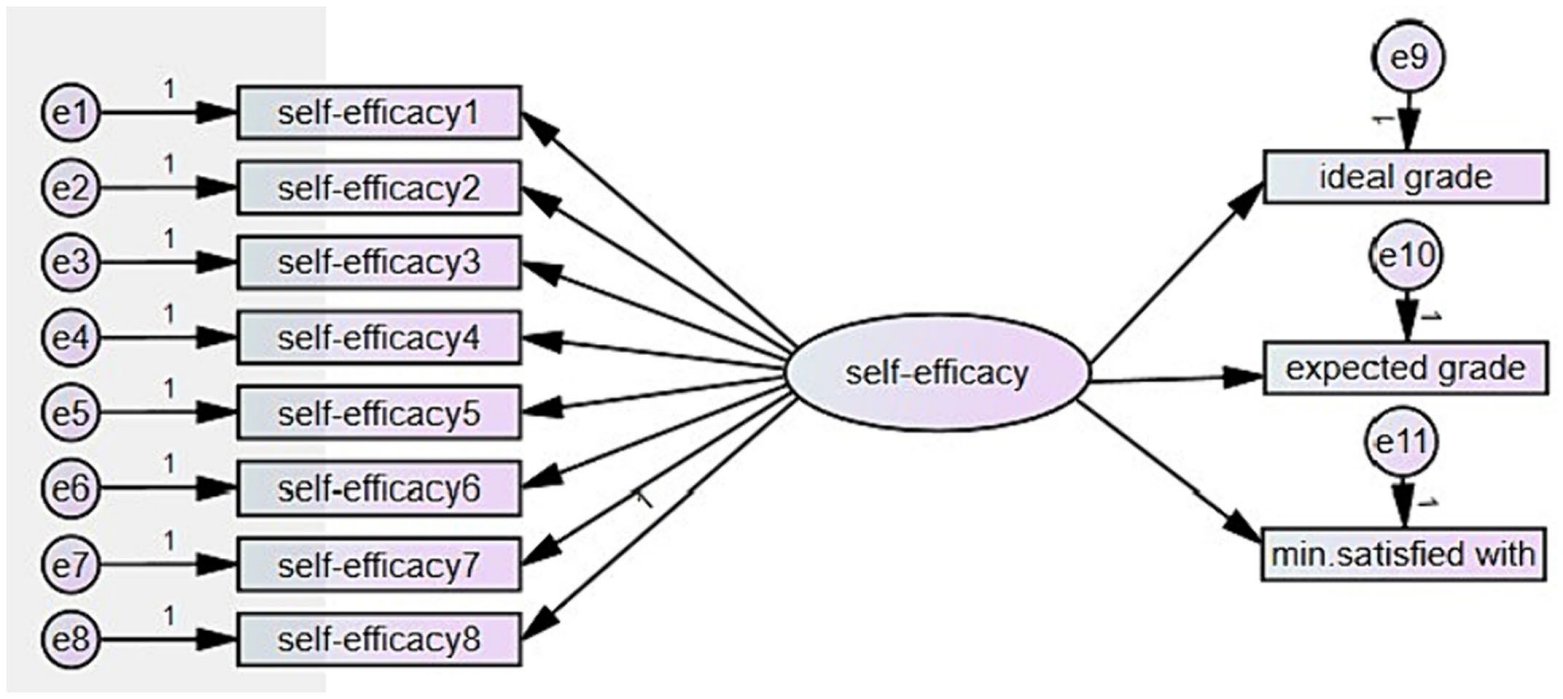
The model of self-efficacy and its positive effect on three self-set grade goals.

When hypothesizing the models, earlier research results were considered. The first model (Figure 1) was created in order to test the relations of self-efficacy measured with MSLQ (Pintrich, 1991) and learners’ three self-set grade goals – ideal, expected, and minimum accepted. According to Zimmerman and Bandura (1994), goals function primarily through self-processes, such as perceived self-efficacy rather than directly controlling motivation and behavioral attainments. Higher self-efficacy leads to higher goals and greater goal commitment. With the first model, it was assumed that self-efficacy has a positive effect on the three self-set grade goals.

**Figure 2:**
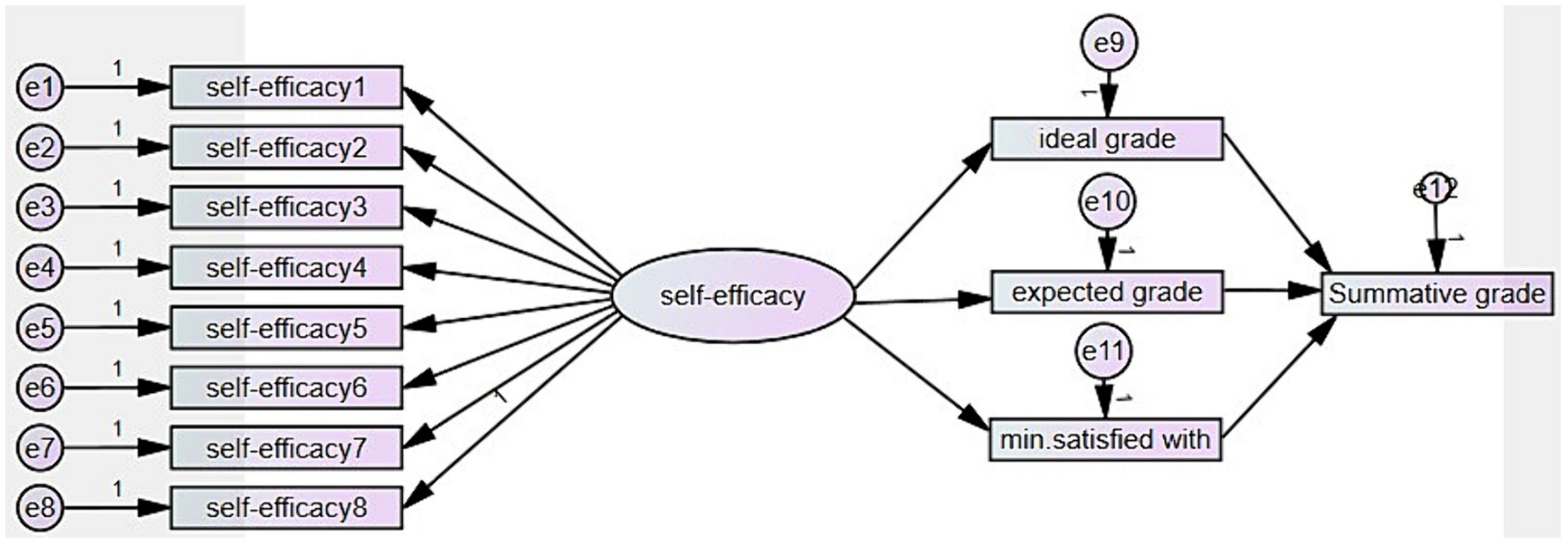
The model of self-set grade goals’ direct effect and self-efficacy’s indirect effect on learning outcome.

With the second model (Figure 2), the direct effect of self-set grade goals and the indirect effect of self-efficacy on learning outcomes (summative course grade) were tested. Self-efficacy has several benefits aside from affecting goal commitment. It does not only predict self-set goals but also performance and retention (Caprara et al., 2008). According to Erez and Judge (2001), self-efficacy influences a person’s choice of goal level, the potency of their tactics, and how they handle failure. In this model, self-set grade goals were considered as a mediator of the effect of self-efficacy on learning outcomes.

Morisano (2013) found that the ideal grade set by the learner tends to be overly optimistic, and the expected grade does not accurately reflect the goal *per se*. Only the minimum grade one would be satisfied with has proven to be the most valid of the self-set grade goals to predict learning outcomes (Locke and Latham, 1990; Morisano, 2013). In order to test the hypotheses, both models were tested and analyzed.

## 3 Method

In order to answer the research questions and test the hypotheses, a quantitative study was conducted as part of the teacher education course Basics of Learning, which is compulsory for all undergraduate and graduate students in the first semester of the first academic year. The fully online course was implemented in the Moodle environment with an introductory face-to-face meeting at the beginning of the course. The syllabus includes eight topics – cognitive development, perception and attention, memory, physical development, self-regulation and metacognition, emotions, and language and speech. Students mostly work independently - listen to video lectures and read materials. Each topic can be discussed in a common discussion forum, and questions are answered in private messages or the public forum. The course has four assignments that are evaluated, and the final grade is formed based on two essays, an observation task on self-regulation carried out in the classroom, and a learning diary where students reflect on their learning experience and analyze their learning process. Student satisfaction with the content of the course and its execution has been relatively high over the years. Most students appreciate the opportunity to work at their own pace, flexibility, and support from the lecturer.

### 3.1 Sample

An invitation to participate in the study was sent to all 353 teacher education students registered for the Basics of Learning online course. Of these, 160 (45%) students answered and completed the self-report questionnaire. Students were not rewarded or incentivized for their participation. 91 (57%) of the respondents were undergraduate students, 69 (43%) were graduates. All students were Caucasian. Approximately 10% of the students were of Russian origin but as the curricula are in Estonian, they study and communicate in Estonian. 50% of the students are working full-time or part-time (undergraduates mostly in kindergartens and vocational education, graduates in schools) and studying simultaneously. As the data collected with the questionnaire were analyzed related to the academic outcomes, the data could not be anonymous. However, the only person who had access to the data and performed the data analysis was the lecturer/the author of the paper. The students were informed about the aim of the study, the procedure of the data collection, data storage, and analysis.

### 3.2 Data collection

Quantitative data on learners’ self-efficacy and self-set grade goals were collected using the LimeSurvey electronic questionnaire. Data on self-efficacy were collected using the MSLQ motivation scale (Pintrich, 1991). The motivation scale has 31 items in six factors (intrinsic goal orientation, extrinsic goal orientation, task value, control of learning beliefs, self-efficacy, and test anxiety), which the respondents rated on the Likert-type scale from 1 – not at all true about me to 7 – very true about me. In addition, respondents were asked to enter three self-set grade goals: the grade they would like to receive for the course (ideal grade), the grade they think they will receive for the course (expected grade), and the minimum grade they would be satisfied with (minimum grade) (Morisano, 2013). Grade goals were entered as letters (A – excellent, B – very good, C – good, D – satisfactory, E – poor, F – failed). After the course, the dataset was supplemented with the learners’ course summative grades (A–F). The grade was formed based on the points obtained for three written assignments (essay, class observation protocol, and short essay) and a reflective learning diary. The maximum score was 27 points.

### 3.3 Data analysis

All data were standardized with *Z*-score normalization before starting the analysis. Standardization was necessary to eliminate the scale differences of the variables. In order to reliably assess respondents’ self-report estimates of their self-efficacy, the factor structure of the motivation scale was tested with confirmatory factor analysis (CFA). Confirmatory factor analysis is a statistical method used to assess the suitability of a set of observed variables (indicators) with a theoretical model. We used maximum likelihood estimation to estimate model parameters—factor loadings, variances, and covariances. Factor loadings indicate the strength of the relationship between each indicator and its corresponding factor. Factors greater than or equal to 0.4 were considered acceptable. The fit of the model to the data was evaluated using goodness-of-fit indices – chi-square test (*χ*^2^), comparative fit index (CFI), Tucker-Lewis index (TLI), and root mean square error of approximation (RMSEA). Fit indices are used to determine whether the model fits the data well or whether the model needs to be modified. Although subsequent analyses continued with only the self-efficacy factor, it was necessary to conduct a CFA on the entire motivation scale to ensure that the items loaded correctly on the factor. CFA was followed by an assessment of factor reliability with Cronbach’s alpha, a measure of internal consistency.

The next step was to analyze the data using descriptive statistics and correlation analysis. The purpose of the latter was to identify relationships between self-efficacy as a predictor, self-set grade goals as a mediator, and summative grade as a dependent variable to create a predictive model. Structural equation modeling was the final step in the data analysis. By combining elements of factor analysis, regression analysis, and path analysis into a single framework, SEM allows for testing and evaluating complex relationships between observed and latent variables. SEM was considered the most appropriate method as it allows the study of both direct and indirect relationships between variables while accounting for measurement error and allowing for the inclusion of latent variables. All analyses were performed using SPSS and AMOS (version 27.0.1.0).

## 4 Results

### 4.1 The psychometric properties and indicators of the factor of self-efficacy (RQ1)

In order to answer the first research question, the factor structure of the whole motivation scale of the MSLQ was tested. As a result of the CFA, most of the items had sufficiently high loadings. The only exceptions were two items in the control of learning beliefs factor, below 0.4 (item 9–0.3; item 25–0.38). After removing these low-loading items, the factor remained with two items. Typically, the smallest acceptable number of items in a factor is three (Tabachnick et al., 2013). In a measurement model, a two-item factor is identifiable when the factor loadings of the items are more or less equal (Bolger et al., 1998). This requirement was unmet (item 2–0.41; item 18–0.68). Another indicator that shows the reliability of the two-point factor is the high correlation between the variables (*r* > 0.70) (Yong and Pearce, 2013). For the current two items, the correlation coefficient was relatively low (*r* = 0.372; *p* < 0.001). Therefore, it was decided to drop the entire factor and the model was identified with five factors (Table 1). The goodness-of-fit indices of the five-factor model were acceptable: *χ*^2^ = 478,358; df = 303; *p* = 0.000; CMIN/DF = 1,578; CFI = 0.919; TLI = 0.906; NFI = 0.810; RMSEA = 0.060. The other psychometric properties are presented in Table 1.

**Table 1:** Cronbach’s alpha coefficients and variance of the factors in the motivation scale.

Factor	Cronbach’s α	Variance	Number of items
Task value	0.89	30.24%	6
Test anxiety	0.78	14.98%	5
Self-efficacy	0.90	8.88%	8
Extrinsic goal orientation	0.73	4.42%	4
Intrinsic goal orientation	0.70	3.99%	4
Total	0.86	62.5%	27

Thus, it can be concluded that the motivation scale of the MSLQ questionnaire (Pintrich, 1991) has good psychometric properties and is reliable for the following analyses.

A correlation analysis was performed in order to understand the relationship between the factors of the motivation scale and the self-set grade goals. The results (Table 2) indicate statistically significant positive and negative, albeit weak correlations between all factors but extrinsic goal orientation and grade goals.

**Table 2:** Correlation coefficients between the factors and self-set grade goals.

	Ideal grade	Expected grade	Minimum accepted
Task value			0.301^**^
Test anxiety	−0.318^**^	−0.238^*^	
Self-efficacy	0.213^*^	0.383^**^	0.431^**^
Extrinsic goal orientation			
Intrinsic goal orientation			0.300^**^

*Correlation is significant at the 0.05 level. **Correlation is significant at the 0.01 level.

The face validity of the scale suggests that the items are clear and unambiguous. The students who completed the questionnaire and later reflected on their perceptions in their learning diaries expressed appreciation for the opportunity to gain deep and meaningful reflection on their self-regulated learning process and analyzed the aspects that need further development. The correlation analysis provided valuable information about the questionnaire factors, indicating strong positive correlations between intrinsic goal orientation, task value, and self-efficacy, which occur together and refer to a learner’s interest and appreciation of the learning content (Taylor, 2012). Another interesting relation appeared between extrinsic goal orientation and test anxiety which are positively correlated with each other but negatively with the other factors. Ideal and expected grades were positively correlated with self-efficacy and negatively with test anxiety. Minimum grade satisfied with was positively correlated with task value, self-efficacy, and intrinsic goal orientation.

Since self-efficacy is the focus of this study, this factor is explored in more detail below. The factor has eight statements (Table 3) loaded to one factor with sufficiently high weights.

**Table 3:** Factor loadings and explained variance (*R*^2^) according to the two dimensions of self-efficacy.

Item	Factor loading	*R* ^2^
*Expectancy for success*		
I believe I will receive an excellent grade in this course.	0.85	0.72
I’m confident I can do an excellent job on the assignments and tests in this course.	0.86	0.74
I expect to do well in this course.	0.71	0.50
Considering the difficulty of this course, the teacher, and my skills, I think I will do well in this course.	0.76	0.58
*Self-efficacy*		
I’m certain I can understand the most difficult material presented in the readings for this course.	0.64	0.41
I’m confident I can understand the basic concepts taught in this course.	0.57	0.32
I’m confident I can understand the most complex material presented by the instructor in this course.	0.58	0.34
I’m certain I can master the skills being taught in this course.	0.61	0.37

Despite the fact that Pintrich (1991) distinguished two separate aspects in the self-efficacy factor – expectancy for success and self-efficacy – in the factor analysis, the two were not distinguished, and in the following analysis, they are treated as one single factor.

According to the learners’ estimates, the average value of their self-efficacy is 4.97 (*SD* = 0.97) (on a 7-point Likert-type scale) with Skewness of −0.64 (StE = 0.19) and Kurtosis of 0.07 (StE = 0.38) which both remain within acceptable limits proving the normal distribution of the data.

### 4.2 The indicators of the learners’ self-set grade goals (RQ2)

**Figure 3:**
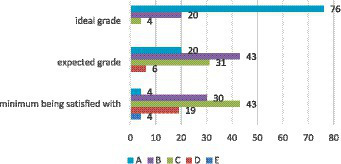
The frequency of learners’ self-set grade goals (%).

In line with the assessment system in the university, the respondents set their grade goals in letters (A – the highest, F – the lowest) which were converted to numbers (A – 5, F – 0) and standardized for further analyses (Figure 3).

Wilcoxon Signed Ranks Test indicated statistically significant differences between the learner’s set goals for the ideal grade (Me = 5) and expected grade (Me = 4): *Z* = −7.758; *p* < 0.001, as well as between expected grade (Me = 4) and the minimum grade being satisfied with (Me = 3): *Z* = −6.861; *p* < 0.001.

### 4.3 The effect of self-efficacy on self-set grade goals – ideal, expected, and minimum acceptable (RQ3)

**Figure 4:**
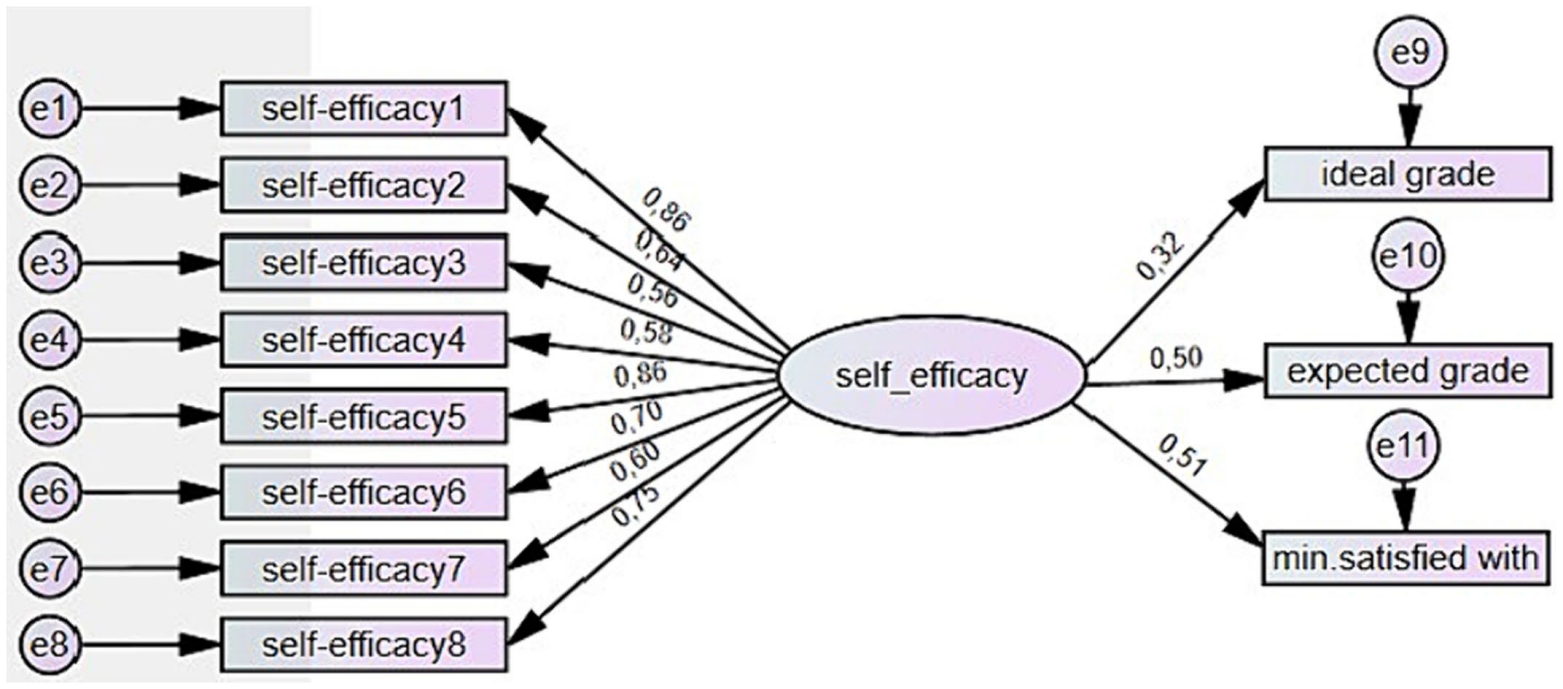
The standardized direct effects of self-efficacy on self-set grade goals.

Correlation analysis was conducted first to assess the effect of self-efficacy on learners’ self-set grade goals. The analysis revealed positive but weak correlations of self-efficacy with two grade goals: ideal grade *r* = 0.213, *p* = 0.034; expected grade *r* = 0.335, *p* = 0.001; minimum being satisfied with *r* = 0.431, *p* = 0.000; and the summative grade *r* = 0.227, *p* = 0.024. These correlations were used as a basis for creating the SEM model. As a result, the Maximum likelihood analysis provided statistically significant effects of self-efficacy on all three grade goals. The standardized path coefficient from self-efficacy to ideal grade (Figure 4) was 0.32 (SE = 0.07, *p* = 0.009), indicating a positive relationship between the two variables. The coefficient was standardized by dividing the path coefficient by the product of the standard deviations of the variables. This effect was, however, smaller than the standardized effect of self-efficacy on expected grade, which was 0.50 (SE = 0.12, *p* = 0.000), or the minimum grade being satisfied with, which was 0.51 (SE = 0.14, *p* = 0.000).

The goodness-of-fit indices of the model indicated acceptable fit: *χ*^2^ = 61,239; df = 38; *p* = 0.010; CMIN/DF = 1,612; CFI = 0.947; TLI = 0.924; NFI = 0.877; RMSEA = 0.079, which proved a positive effect of self-efficacy on learners’ grade goals. Thus, the first hypothesis on the positive effect of self-efficacy on learner self-set grade goals was confirmed.

### 4.4 The effect of self-set grade goals – ideal, expected, and minimum acceptable – mediating self-efficacy and actual learning outcomes (RQ4)

**Figure 5:**
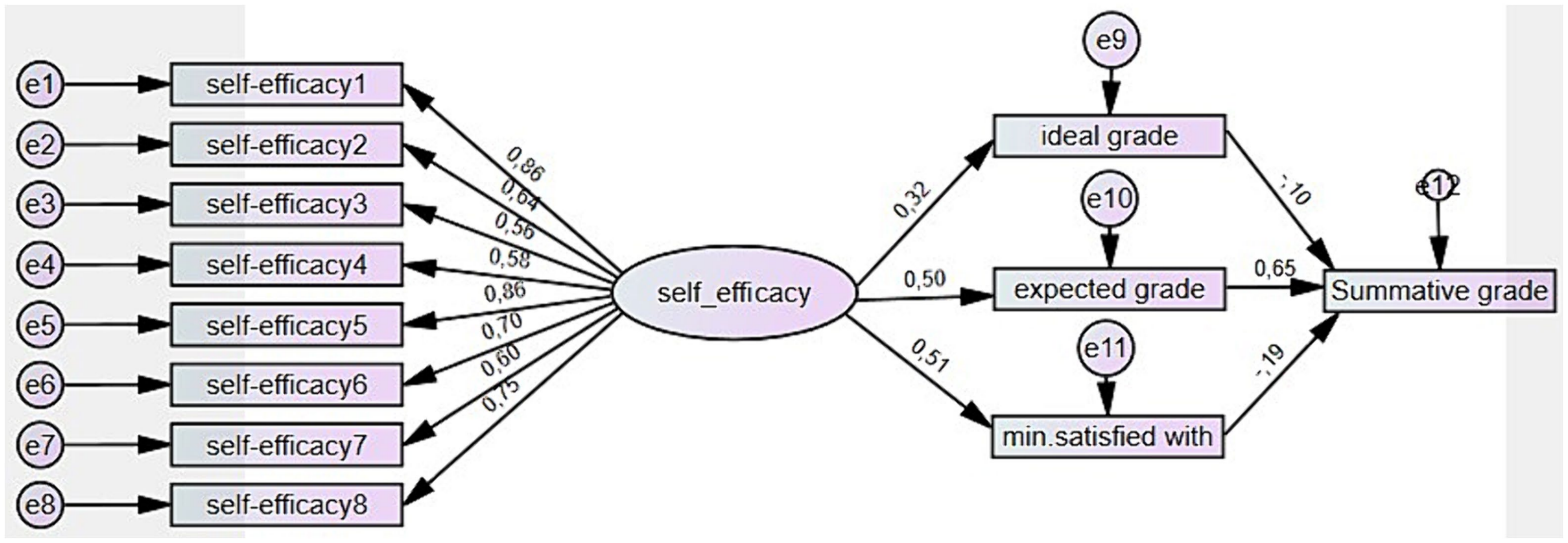
The standardized direct and indirect effects of self-efficacy and self-set grade goals on learning outcomes.

After making the model more complex by adding the summative course grade, the coefficients of the mediators’ effects changed (Figure 5).

**Figure 6:**
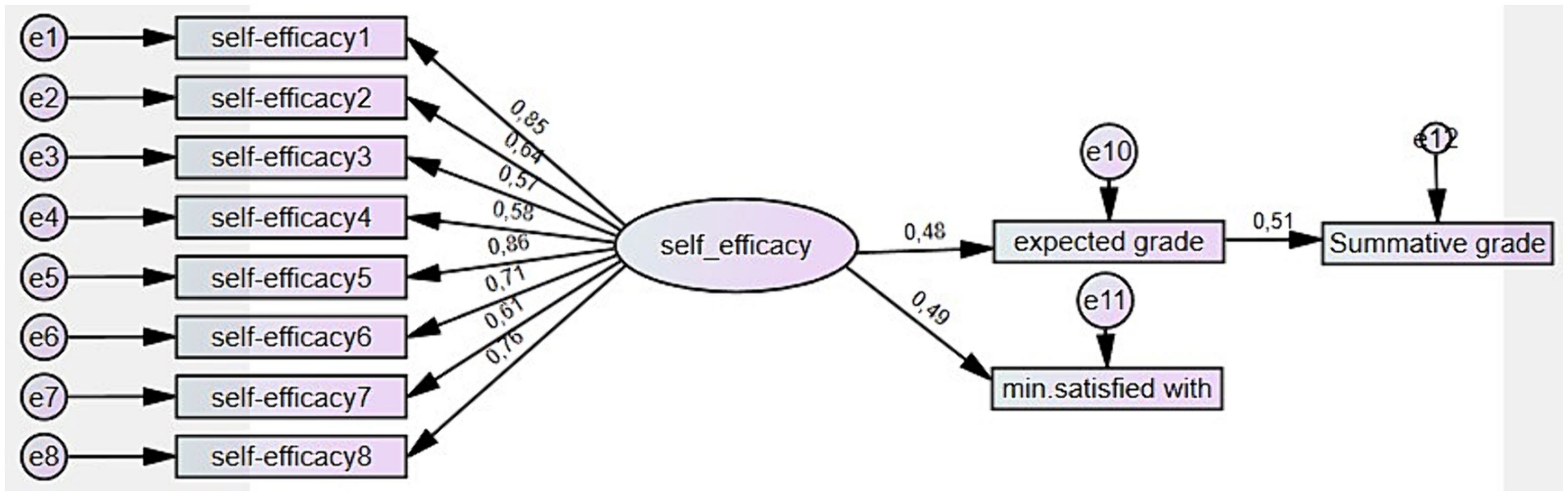
Standardized direct effects of self-efficacy and self-set grade goals.

The ideal grade goal was no longer statistically significant and was therefore removed from the model (Figure 6). The expected grade goal showed a significant mediating effect of self-efficacy on learning outcomes. The standardized effect of self-efficacy on expected grade was 0.48 (SE = 0.15, *p* = 0.000), and on minimum satisfied with 0.49 (SE = 0.15, *p* = 0.000). The expected grade is the only learners’ self-set grade goal which predicts the summative course grade – standardized effect 0.51 (SE = 0.09, *p* = 0.000). The standardized indirect effect of self-efficacy on the summative course grade is 0.25.

The goodness-of-fit indices of the model indicated good fit: *χ*^2^ = 52,144; df = 40; *p* = 0.045; CMIN/DF = 1,304; CFI = 0.971; TLI = 0.960; NFI = 0.891; RMSEA = 0.060, which shows that the model fits well with the observed data and that the effect sizes reliably predict the course grade. Thus, the second hypothesis, which stated that the minimum grade is the most accurate predictor of the actual learning outcome, was rejected. The third hypothesis about the indirect effect of self-efficacy on learning outcomes was confirmed.

## 5 Discussion

Self-processes are established as essential for self-regulated learning, goal setting, and learning outcomes in several motivational research. However, research on the connection between goal setting and self-efficacy and their effects on academic accomplishment has been contradictory. This study aimed to assess the influence of self-efficacy and self-set grade goals on academic outcomes. In order to address the research questions, an exploratory study was carried out with teacher training students from a higher education institution within the context of an online course.

The MSLQ motivation scale (Pintrich, 1991) retained five factors due to model estimation. Its acceptable model fit indices confirmed the consistency of the theoretical model with our observed data. The correlation analysis indicated the relationship between several motivational factors and self-set grade goals. The most telling of these is connected with test anxiety and its negative correlations with self-efficacy and grade goals. It has previously been detected that academic stress affects students’ perceptions of their grade goals and expectations (Tan et al., 2008), creating significant barriers to learning and performance (Andrews and Wilding, 2004) and leading to lower outcomes and higher student drop-out rates (Vaez and Laflamme, 2008). Although some anxiety is normal and often helps maintain mental and physical alertness, excessive anxiety should be controlled by teaching how to mitigate test-taking skill deficits by combining skill-focused strategies with cognitive and behavioral approaches (Ergene, 2003). Another interesting correlation appeared between minimum grade satisfied with, task value and intrinsic goal orientation. The latter two have also revealed relatedness in earlier research findings (e.g., Khan and Khan, 2015), confirming the role of motivational factors on goal orientation.

Similar to the latest validity studies (e.g., Maison and Syamsurizal, 2019), incl. in the context of e-learning (e.g., Khosim and Awang, 2020), this study also confirmed a single factor that includes the two aspects of expectancy for success and self-efficacy. Although some studies (e.g., Lee et al., 2020) have identified the items of these two aspects as different factors, the current sample of teacher training students’ performance expectations, i.e., their perceptions and beliefs about academic achievement, were strongly related to their self-appraisal of their ability to understand course materials. Greater concordance between expectancy and self-efficacy may result from adult learners’ more realistic self-view as a learner and more determined attitude toward their learning process. This is also confirmed by the results of the correlation analysis, which indicate a connection between self-efficacy and self-set grade goals.

The connectedness of self-efficacy and goals has been reported since the early works of goal setting (Locke and Latham, 1990) and efficacy (Bandura, 1997). Higher self-efficacy leads to higher goals and a stronger commitment to goals (Alhadabi and Karpinski, 2020). Although the present study did not examine goal commitment, it clearly showed a direct effect of self-efficacy on all self-set grade goals, the highest on expected grade and minimum being satisfied with. It refers to the adult learners’ realistic performance expectations, setting goals that are attainable (Brunstein, 1993) and relatively close at hand (Koestner et al., 2002), plus realistic self-esteem. This conclusion is supported by the fact that the grade goal, which most accurately predicts the learning outcome, is the expected grade based on the student’s self-assessment of which grade they are most likely to receive.

What goals the learners set for themselves depends predominantly on their academic self-efficacy assessments. As mentioned above, the learner’s self-efficacy is based on various motivational factors. Given that the participants in the present study set their grade goals in the context of the entire course (rather than for individual assignments or tasks), it can be inferred that outcome expectations influenced their decisions directly, whereas efficacy expectations, which also considered their past effort, persistence, and learning strategies, had an indirect effect on their learning outcomes. In contrast to previous research that found the minimum grade to be the most accurate measure of grade goal (Bertrams, 2012; Morisano, 2013), this study found the expected grade to be the most precise predictor of academic performance. The reason for the difference in results can be caused by the sample, which in Morisano’s study consisted of academically struggling students, while in our case, the sample was partly made of young people starting their undergraduate studies in the teacher training curricula, and partly of working teachers who need to improve their professional qualifications (however, no significant differences were revealed in comparing these two different sample groups). Their self-efficacy beliefs may differ, but the latter’s also more realistic and accurate, as shown by the higher predictive power of the expected grade goal. We can also hope the adult learner’s self-concept is good enough to set adequate, relevant, and achievable goals. On the other hand, motivational factors can also play an important role in setting goals. Starting from the reasons why one comes to study the chosen study program to the feelings and expectations of enrolling in the chosen (albeit mandatory) subject course.

The main limitation of the study is related to the sample. The relatively small number of respondents did not allow for invariance analyses or comparisons between groups of graduate and undergraduate or working and non-working students. The fact that they all come from the same institution but with different curricula does not allow the results to be generalized to wider student populations; they can only be interpreted in this context.

This article extends previous research and contributes to the existing literature on the relationships between self-efficacy, self-set grade goals, and learning outcomes. There is a paucity of published research on this topic highlighting the influence of motivational factors in online learning, and this study fills this particular gap by providing insight into the predictive effects of self-efficacy and grade goals. Studies (e.g., Morisano, 2013) have shown the mediating and moderating effects of several different factors. This research showed the importance of self-efficacy when setting realistic goals for one’s learning process based on one’s perception of one’s ability and resources. Self-efficacy, which is crucial for academic success and general development, can be supported by teachers in various learning situations. It is important to ensure that students understand the expectations and learning objectives. Learners are more likely to have confidence in their capacity to meet expectations when they know what is expected of them. Assisting students in establishing reasonable, attainable goals and breaking difficult tasks into smaller, more doable ones increases their confidence and self-efficacy. Students should understand the importance of effort and hard work in the learning process, the idea that challenges and setbacks are normal, and that learning often involves overcoming obstacles. The classroom environment where students feel safe to take risks and make mistakes, collaboration, and peer support help create a learning community that values every student’s individual contribution. No less important is constructive feedback that focuses on effort, progress, and improvement rather than the final outcome. Students should be encouraged to reflect on their own learning experiences. Reflection helps them become more aware of their strengths and areas for improvement, fostering a sense of control over their learning, autonomy, and agency. A supportive and encouraging teacher-student relationship and learning atmosphere greatly contribute to students’ self-efficacy and encourage them to set and strive for higher goals. The future research will consider the effects of effort, persistence, and commitment on both goal setting and learning outcomes. We also want to evaluate the motivation factors for different sample groups, considering their status in the labor market and their motives for starting to study. Further research needs to reach larger samples to include additional variables such as undergraduate/graduate academic status or working/non-working status.

## Data availability statement

The original contributions presented in the study are included in the article/supplementary materials, further inquiries can be directed to the corresponding author.

## Ethics statement

The requirement for ethical approval was waived based on the requirements of the University of Tartu. The study was conducted in accordance with local legislation and institutional requirements. Participants gave their written informed consent to participate in this study.

## Author contributions

KS: Conceptualization, Data curation, Formal analysis, Investigation, Methodology, Resources, Validation, Visualization, Writing – original draft, Writing – review & editing.
